# A mathematical model of p62-ubiquitin aggregates in autophagy

**DOI:** 10.1007/s00285-021-01659-2

**Published:** 2021-12-14

**Authors:** Julia Delacour, Marie Doumic, Sascha Martens, Christian Schmeiser, Gabriele Zaffagnini

**Affiliations:** 1 Inria, CNRS, Laboratoire Jacques-Louis Lions, Sorbonne Université, Université Paris-Diderot, 75005 Paris, France; 2 Max F. Perutz Laboratories Vienna Biocenter (VBC), University of Vienna, Dr. Bohr-Gasse 9, 1030 Vienna, Austria; 3 Faculty of Mathematics, University of Vienna, Oskar-Morgenstern-Platz 1, 1090 Vienna, Austria; 4 Centre for Genomic Regulation (CRG), Dr. Aiguader 88, 08003 Barcelona, Spain

**Keywords:** 92C40 Biochemistry, molecular biology

## Abstract

Aggregation of ubiquitinated cargo by oligomers of the protein p62 is an important preparatory step in cellular autophagy. In this work a mathematical model for the dynamics of these heterogeneous aggregates in the form of a system of ordinary differential equations is derived and analyzed. Three different parameter regimes are identified, where either aggregates are unstable, or their size saturates at a finite value, or their size grows indefinitely as long as free particles are abundant. The boundaries of these regimes as well as the finite size in the second case can be computed explicitly. The growth in the third case (quadratic in time) can also be made explicit by formal asymptotic methods. In the absence of rigorous results the dynamic stability of these structures has been investigated by numerical simulations. A comparison with recent experimental results permits a partial parametrization of the model.

## Introduction

Autophagy is an intracellular pathway, which targets damaged, surplus, and harmful cytoplasmic material for degradation. This is mediated by the sequestration of cytoplasmic cargo material within double membrane vesicles termed autophagosomes, which subsequently fuse with lysosomes wherein the cargo is hydrolyzed. Defects in autophagy result in various diseases including neurodegeneration, cancer, and uncontrolled infections (Levine and Kroemer 2008). The selectivity of autophagic processes is mediated by cargo receptors such as p62 (also known as SQSTM1), which link the cargo material to the nascent autophagosomal membrane (Danieli and Martens 2018). p62 is an oligomeric protein and mediates the selective degradation of ubiquitinated proteins. Its interaction with ubiquitin is mediated by its C-terminal UBA domain, while it attaches the cargo to the autophagosomal membrane due to its interaction with Atg8 family proteins such as LC3B, which decorate the membrane (Pankiv et al. 2007). Additionally, p62 serves to condensate ubiquitinated proteins into larger condensates or aggregates, which subsequently become targets for autophagy (Sun et al. 2018; Zaffagnini et al. 2018). It has been reported that this condensation reaction requires the ability of p62 to oligomerize and the presence of two or more ubiquitin chains on the substrates (Wurzer et al. 2015; Zaffagnini et al. 2018).

In this article a mathematical model for the condensation process is derived and analyzed. It is based on cross-linking of p62 oligomers by ubiquitinated substrate (Zaffagnini et al. 2018). A cross-linker is assumed to be able to connect two oligomers, where each oligomer has a number of binding sites corresponding to its size. As an approximation for the dynamics of large aggregates, a nonlinear system of ordinary differential equations is derived.

The oligomerization property of p62 has been shown to be necessary in the formation of aggregates (Zaffagnini et al. 2018): too small oligomers of Ubiquitin do not form aggregates (Wurzer et al. 2015).

The dynamics of protein aggregation has been studied by mathematical modelling for several decades, but most models consider the aggregation of only one type of protein, which gives rise to models belonging to the class of nucleation–coagulation–fragmentation equations, see e.g. Bishop and Ferrone (1984), Prigent et al. (2012) and Xue et al. (2008) for examples in the biophysical literature, and Collet et al. (2002), Laurençot and Mischler (2002), Banasiak and Lamb (2006) and Dubovskiĭ and Stewart (1996) for a sample of the mathematical literature. Contrary to these studies, the present work considers aggregates composed of two different types of particles with varying mixing ratios, which drastically increases the complexity of the problem.

In the following section the mathematical model is derived. It describes an aggregate by three numbers: the number of p62 oligomers, the number of cross-linkers bound to one oligomer, and the number of cross-linkers bound to two oligomers. The model considers an early stage of the aggregation process where the supply of free p62 oligomers and of free cross-linkers is not limiting. Since no other information about the composition of the aggregate is used, assumptions on the binding and unbinding rates are necessary. In the limit of large aggregates, whose details are presented in an appendix, the model takes the form of a system of three ordinary differential equations. Section 3 starts with a result on the well posedness of the model, and it is mainly devoted to a study of the long-time behaviour by a combination of analytical and numerical methods. Depending on the parameter values, three different regimes are identified, where either aggregates are unstable and completely dissolved, or their size tends to a limiting value, or they keep growing (as long as they do not run out of free oligomers and cross-linkers). In Sect. 4 we discuss the parametrization of the model and a comparison with data from Zaffagnini et al. (2018).

## Presentation of the model

### Discrete description of aggregates

We consider two types of basic particles:

1. *Oligomers* of the protein p62, where we assume for simplicity that all oligomers contain the same number $n\ge 3$ of molecules (see Sect. 4 for a discussion of this assumption). These oligomers are denoted by p62$_n$ and are assumed to possess *n* binding sites for ubiquitin each,
2. *Cross-linkers* in the form of ubiquitinated cargo, denoted by *Ubi* and assumed to have two ubiquitin ends each. When one end of a *Ubi* is bound to a p62$_n$, we call it *one-hand bound,* when both ends are bound we call it *both-hand bound.*

An aggregate is represented by a triplet $(i,j,k)\in {\mathbb {N}}_0^3$, where *i* denotes the number of one-hand bound *Ubi*, *j* denotes the number of both-hand bound *Ubi*, and *k* denotes the number of p62$_n$. An aggregate will be assumed to contain at least two p62$_n$, i.e. $k\ge 2$, and enough both-hand bound *Ubi* to be connected, i.e. $j\ge k-1$. Furthermore, an aggregate contains *nk* binding sites for *Ubi*, implying $i+2j \le nk$. A triplet $(i,j,k)\in {\mathbb {N}}_0^3$ satisfying the three inequalities

1 $$\begin{aligned} k\ge 2 ,\qquad j\ge k-1 ,\qquad i+2j \le nk , \end{aligned}$$

will be called *admissible.* It is a rather drastic step to describe an aggregate only by these three numbers, since the same triplet might represent aggregates with various shapes. An example of an admissible triplet describing a unique aggregate topology is $(0,k-1,k)$, representing a chain of p62$_n$. Adding one both-hand bound *Ubi* already creates a topological ambiguity: The triplet (0, *k*, *k*) can be realized by a circular aggregate or by an open chain, where one connection is doubled. Apart from missing topological information, the triplet (*i*, *j*, *k*) also lacks any information on the geometry of an aggregate. The reaction rates described below should be interpreted as mean values taken over all possible aggregate shapes described by a triplet (*i*, *j*, *k*). An extension of the model to include the shape information would require knowledge on the conformations of oligomers and cross-linkers, including chemical and mechanical properties, which is only partially available in the literature [see, e.g., Ciuffa et al. (2015) for p62 oligomers]. The modeling of aggregation reactions would then require molecular dynamics simulations, which would be rather complex even for small aggregates. For our goal of describing the dynamics of large aggregates such an approach seems to be prohibitively complex.

### The reaction scheme

Basically there are only two types of reactions: binding and unbinding of *Ubi* to p62$_n$. However, depending on the situation these may have various effects on the aggregate, whence we distinguish between three binding and three unbinding reactions.

1. **Addition of a free** *Ubi*, requiring at least one free binding site, i.e. $nk-i-2j\ge 1$, (see Fig. 1):
  $$\begin{aligned} Ubi+ (i,j,k) \xrightarrow {\kappa _1'}(i+1,j,k) \end{aligned}$$
  The reaction rate (number of reactions per time) is modeled by mass action kinetics for a second-order reaction with reaction constant $\kappa _1'$ and with the number [*Ubi*] of free *Ubi*. Since free *Ubi* and free p62 oligomers will be assumed abundant, their numbers [*Ubi*] and $[\mathrm{p62}_n]$ will be kept fixed and the abbreviation $\kappa _1 = \kappa _1' [Ubi]$ will be used (see Sect. 5 for a short discussion of the effect of lifting the abundancy assumption). This leads to a first-order reaction rate
  2 $$\begin{aligned} r_1 = \kappa _1 (nk-i-2j) . \end{aligned}$$
2. **Addition of a free** p62$_n$, requiring at least one one-hand bound *Ubi*, i.e. $i\ge 1$:
  $$\begin{aligned} \mathrm{p62}_n+ (i,j,k) \xrightarrow {\kappa _2'}(i-1,j+1,k+1) \end{aligned}$$
  Analogously to above, we set $\kappa _2 = \kappa _2' [\mathrm{p62}_n]$ and
  3 $$\begin{aligned} r_2 = \kappa _2 i . \end{aligned}$$

**Fig. 1:**
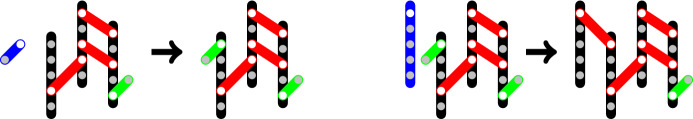
Examples for Reactions 1 (left) and 2 (right) with p62$_5$ in black, one-hand bound *Ubi* in green, two-hand bound *Ubi* in red, free particles in blue. Reaction 1: $Ubi + (1,3,3) \rightarrow (2,3,3)$. Reaction 2: p62$_5 + (2,3,3) \rightarrow (1,4,4)$
3. **Compactification of the aggregate** by a *Ubi* binding its second hand, requiring at least one one-hand bound *Ubi*, i.e. $i\ge 1$, and at least one free binding site, i.e. $nk-i-2j\ge 1$:
  $$\begin{aligned} (i,j,k) \xrightarrow {\kappa _3'} (i-1,j+1,k) \end{aligned}$$
  This is a second-order reaction with rate
  4 $$\begin{aligned} r_3 = \kappa _3' i(nk-i-2j) . \end{aligned}$$
4. **Loss of a** *Ubi*, requiring at least one one-handed *Ubi*, i.e. $i\ge 1$. This is the reverse reaction to 1:
  $$\begin{aligned} (i,j,k)&\xrightarrow {\kappa _{-1}} Ubi + (i-1,j,k) \end{aligned}$$
  Its rate is modeled by
  5 $$\begin{aligned} r_{-1} = \kappa _{-1} i . \end{aligned}$$
5. **Loss of a** p62$_n$ (leading to loss of the whole aggregate if $k=2$):
  $$\begin{aligned} (i,j,k)&\xrightarrow {\kappa _- \alpha _{j,k}} \mathrm{p62}_n + \ell \,Ubi + (i+1-\ell ,j-1,k-1) \end{aligned}$$
  This and the following reaction need some comments. They are actually both the same reaction, namely breaking of a cross-link, which we assume to occur with rate $\kappa _- j$. However, this can have different consequences. Here we consider something close to the reverse of reaction 2. This means we assume that the broken cross-link has been the only connection of a p62 oligomer with the aggregate, such that the oligomer falls off. This requires the condition $nk-2j\ge n-1$, meaning the possibility that the other $n-1$ binding sites of the lost oligomer are free of two-hand bound *Ubi*. It is not quite the reverse of reaction 2, since we have to consider the possibility that $\ell$ one-hand bound *Ubi*, $0\le \ell \le n-1$, are bound to the lost oligomer. The conditional probability $\alpha _{j,k}$ to be in this case, when a cross-link breaks, is *zero* for a very tightly connected aggregate where each oligomer is cross-linked at least twice, i.e. $nk-2j \le n-2$, and it is *one* for a very loose aggregate, i.e. a chain with $j=k-1$. This motivates the model
  6 $$\begin{aligned} \alpha _{j,k} = \frac{(nk-2j-n+2)_+}{(n-2)k+4-n} , \end{aligned}$$
  which is, as a function of $j\in [k-1, (nk-n+2)/2]$, the linear interpolant between the values 1 and 0; notation: $a_+ = \max \{a,0\}$, leading to the rate
  7 $$\begin{aligned} r_{-2} = \kappa _- \alpha _{j,k} j . \end{aligned}$$
  In the framework of our model, $\ell$ should be a random number satisfying the restrictions
  8 $$\begin{aligned} (n-1-nk+i+2j)_+ \le \ell \le \min \{i,n-1\} , \end{aligned}$$
  where the upper bound should be obvious and the lower bound implies that the last condition in (1) is satisfied by the post-reaction state $(i+1-\ell ,j-1,k-1)$. We shall use the choice
  9 $$\begin{aligned} \ell = \ell _{i,j,k} := \left\lfloor \frac{(n-1)i}{nk-2j} \right\rceil , \end{aligned}$$
  which can be interpreted as the rounded ($\lfloor \cdot \rceil$ denotes the closest integer) expectation value for the number of one-hand bound *Ubi* on the lost oligomer in terms of the ratio between the number $n-1$ of available binding sites on the lost oligomer and the total number $nk-2j$ of available binding sites for one-hand bound *Ubi* in the whole aggregate. It is easily seen that in the relevant situation $\alpha _{j,k}>0$, i.e. $nk-2j\ge n-1$, the choice (9) without the rounding satisfies the conditions (8). Since the bounds in (8) are integer, the same is true for the rounded version.
  Note that we neglect the possibility to lose more than one oligomer by breaking a cross-link, i.e. the fragmentation of the aggregate into two smaller ones. On the one hand, this is a serious and actually questionable modeling assumption. Dropping it, on the other hand, would induce serious difficulties in the further development of our model. First, a decision would be required, which of the two post-fragmentation aggregates to follow. Second and more importantly, it would mean to allow for large jumps in the state of the aggregate, ruling out a differential equation model (as derived below), which is based on gradual state changes. An a posteriori justification of the no-fragmentation assumption will be provided by some of the results of the following section, showing that growing aggregates are tightly connected, making fragmentation very unlikely (Fig. 2).
6. **Loosening of the aggregate** by breaking a cross-link, requiring at least one excess both-hand bound *Ubi*, i.e. $j\ge k$:
  $$\begin{aligned} (i,j,k)&\xrightarrow {\kappa _- (1-\alpha _{j,k})} (i+1,j-1,k) . \end{aligned}$$
  This is the reverse of reaction 3 with the rate
  10 $$\begin{aligned} r_{-3} = \kappa _- (1-\alpha _{j,k})j , \end{aligned}$$
  which respects the requirement $j\ge k$ for a positive rate, because of
  $$\begin{aligned} 1 - \alpha _{j,k} = \min \left\{ 1, \frac{2(j-k+1)}{(n-2)k+4-n} \right\} . \end{aligned}$$

**Fig. 2:**
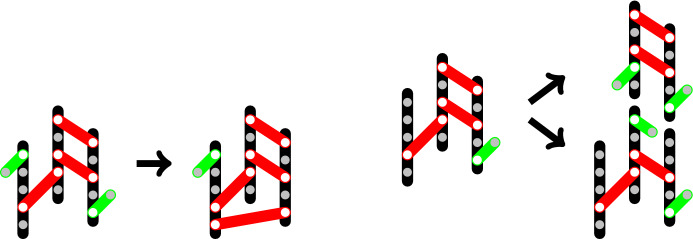
Examples for Reaction 3 (left, $(2,3,3)\rightarrow (1,4,3)$), Reaction 5 (right, up, $(1,3,3)\rightarrow \mathrm{p62}_5 + (2,2,2)$, $\ell =0$), and Reaction 6 (right, down, $(1,3,3)\rightarrow (2,2,3)$)

### A deterministic model for large aggregates

The next step is the formulation of an evolution problem for a probability density on the set of admissible states (*i*, *j*, *k*). In this problem the discrete state is scaled by a typical value $k_0$ of $\kappa _1/\kappa _3'$ and $\kappa _2/\kappa _3'$, assumed of the same order of magnitude. We also assume $k_0$ to be large, which means that we assume the concentrations [*Ubi*] and $[\mathrm{p62}_n]$ of free particles to be large enough (see Sect. 4 for a further discussion of these assumptions):

11 $$\begin{aligned} p := \frac{i}{k_0} ,\qquad q := \frac{j}{k_0} ,\qquad r := \frac{k}{k_0} . \end{aligned}$$

This choice means that the orders of magnitude $\kappa _1 k_0$, $\kappa _2 k_0$, and $\kappa _3' k_0^2$ of the forward reaction rates (2), (3), (4) are the same. It is then consistent with these definitions to introduce $\kappa _3 := \kappa _3' k_0$ and to assume that $\kappa _3$ takes moderate values. In the large aggregate limit $k_0\rightarrow \infty$, the new unknowns become continuous, and the equation for the probability density becomes a transport equation (see “Appendix A” for the details). It possesses deterministic solutions governed by the ODE initial value problem

12 $$\begin{aligned} \begin{array}{llll} \dot{p} = (\kappa _1 - \kappa _3 p) (nr-p-2q) + \kappa _{-} q\left( 1- \frac{(n-1)p}{(n-2)r}\right) - (\kappa _2 + \kappa _{-1})p , &{}\quad p(0)=p_0 ,\\ \dot{q} = \kappa _2 p + \kappa _3 p(nr-p-2q) - \kappa _{-}q , &{}\quad q(0)=q_0 , \\ \dot{r} = \kappa _2 p - \kappa _{-} q \alpha (q,r) , &{}\quad r(0)=r_0, \end{array} \end{aligned}$$

where

13 $$\begin{aligned} \alpha (q,r):=\frac{nr-2q}{(n-2)r} \end{aligned}$$

is the limit of $\alpha _{j,k}$ as $k_0\rightarrow \infty$. The conditions for admissible states $(p,q,r)\in [0,\infty )^2\times (0,\infty )$ are obtained in the limit of (1):

14 $$\begin{aligned} s:= nr-p-2q \ge 0 ,\qquad q \ge r , \end{aligned}$$

implying, as expected,

15 $$\begin{aligned} 0 \le \alpha (q,r) \le 1 . \end{aligned}$$

The equations satisfied by *s* and $q-r$,

16 $$\begin{aligned} \dot{s}= & {} (n-1)\kappa _2 p +\kappa _{-1} p +\kappa _{-} q \frac{2(q-r)}{(n-2)r} - s\left( \kappa _3 p + \kappa _1 + \kappa _{-} q \frac{n-1}{(n-2)r}\right) , \end{aligned}$$

17 $$\begin{aligned} (q-r)\dot{\,}= & {} \kappa _3 p s - \frac{2\kappa _- q}{(n-2)r}(q-r) , \end{aligned}$$

show that the conditions (14) are propagated by (12).

## Analytic results

### Global existence

Since the right hand sides of (12) contain quadratic nonlinearities, it seems possible that solutions blow up in finite time. On the other hand, the right hand sides are not well defined for $r=0$. The essence of the following global existence result is that neither of these difficulties occurs.

#### Theorem 1

Let $3\le n\in {\mathbb {N}}$ and $\kappa _1,\kappa _2,\kappa _3,\kappa _{-1},\kappa _{-} \ge 0$. Let $(p_0,q_0,r_0) \in (0,\infty )^3$ satisfy (14). Then problem (12) has a unique global solution satisfying $(p(t),q(t),r(t))\in (0,\infty )^3$ as well as (14) for any $t>0$. Also the following estimates hold for $t>0$:

18 $$\begin{aligned}&p(t)+ q(t)+r(t) \le (p_0+q_0+r_0) \exp \left( t\,\max \{\kappa _1 n,\kappa _2\}\right) , \end{aligned}$$

19 $$\begin{aligned}&r(t) \ge \frac{2}{n}q(t) \ge \frac{2q_0}{n} \exp (-\kappa _{-} t) . \end{aligned}$$

#### Proof

Local existence and uniqueness is a consequence of the Picard–Lindelöf theorem. Global existence will follow from the bounds stated in the theorem. Positivity of the solution components, of $s=nr-p-2q$, and of $q-r$ is an immediate consequence of the form of the equations (12), (16), (17). This also implies

$$\begin{aligned} \dot{p} + \dot{q} + \dot{r} \le \kappa _1 nr + \kappa _2 p \le \max \{\kappa _1 n, \kappa _2\}(p+q+r) , \end{aligned}$$

which shows (18) by the Gronwall lemma. With (14), the equation for *q* in (12) implies

$$\begin{aligned} \dot{q} \ge - \kappa _- q , \end{aligned}$$

and another application of the Gronwall lemma and of (14) proves (19) and, thus, completes the proof of the theorem. $\square$

### Long-time behaviour

The first step in the long-time analysis is the investigation of steady states. Although the right hand sides of (12) are not well defined for $r=0$, the origin $p=q=r=0$ can be considered as a steady state since

$$\begin{aligned} 0\le \alpha (q,r)\le 1 \qquad \text{ and }\qquad \frac{p}{r} \le n \end{aligned}$$

hold for admissible states satisfying (14) and, thus, the right hand sides of (12) tend to zero for admissible $(p,q,r)\rightarrow (0,0,0)$. The following result shows that at most one other steady state is possible which, somewhat miraculously, can be computed explicitly.

#### Theorem 2

Let $3\le n\in {\mathbb {N}}$, $\kappa _1,\kappa _2,\kappa _3,\kappa _{-1},\kappa _- >0$, and let

20 $$\begin{aligned} {\bar{\alpha }} := \frac{n}{n-2} + \frac{\kappa _{-1} + \kappa _1 - \sqrt{(\kappa _1 + \kappa _{-1})^2 + 4 \kappa _1 \kappa _2 (n-1)}}{\kappa _{-} (n-1)} \end{aligned}$$

satisfy $0<{\bar{\alpha }} <1$. Then there exists an admissible steady state $({\bar{p}},{\bar{q}},{\bar{r}})\in (0,\infty )^3$ of (12) given by

$$\begin{aligned} {\bar{p}}= & {} \frac{\kappa _1 \kappa _2 (n-2)}{\kappa _3 (\kappa _- {\hat{q}} (n-1) + \kappa _{-1} (n-2))}\, \frac{1-{\bar{\alpha }}}{{\bar{\alpha }}} ,\\ {\bar{q}}= & {} \frac{\kappa _1 \kappa _2^2 (n-2)}{\kappa _3 \kappa _-(\kappa _- {\hat{q}} (n-1) + \kappa _{-1} (n-2))}\, \frac{1-{\bar{\alpha }}}{{\bar{\alpha }}^2} ,\\ {\bar{r}}= & {} \frac{\kappa _1 \kappa _2^2 (n-2)}{{\hat{q}}\kappa _3 \kappa _-(\kappa _- {\hat{q}} (n-1) + \kappa _{-1} (n-2))}\, \frac{1-{\bar{\alpha }}}{{\bar{\alpha }}^2} ,\\ \end{aligned}$$

with ${\bar{\alpha }} = \alpha ({\bar{q}},{\bar{r}})$ and ${\hat{q}} = (n - (n-2){\bar{\alpha }})/2 \in (1,n/2)$. There exists no other steady state (besides the origin).

#### Proof

The origin is the only steady state with $r=0$, since by (14), i.e. $p+2q \le nr$, $r=0$ implies $p=q=0$. Assuming ${\bar{r}}> 0,$ we introduce

21 $$\begin{aligned} {\hat{p}} = \frac{{\bar{p}}}{{\bar{r}}} , \qquad \qquad {\hat{q}} = \frac{{\bar{q}}}{{\bar{r}}} , \end{aligned}$$

and rewrite the steady state equations in terms of ${\hat{p}}$ and ${\hat{q}}$:

22 $$\begin{aligned} 0&= (\kappa _1-\kappa _3 {\bar{p}})(n-{\hat{p}}-2{\hat{q}}) + \kappa _{-} {\hat{q}} \left( 1 - {\hat{p}}\frac{n-1}{n-2}\right) -(\kappa _2 + \kappa _{-1}){\hat{p}}, \end{aligned}$$

23 $$\begin{aligned} 0&= \kappa _2 {\hat{p}} + \kappa _3 {\bar{p}} (n-{\hat{p}}-2{\hat{q}}) -\kappa _{-} {\hat{q}}, \end{aligned}$$

24 $$\begin{aligned} 0&= \kappa _2 {\hat{p}} -\kappa _{-} {\hat{q}} {\bar{\alpha }},\quad \text{ with } {\bar{\alpha }}=\frac{n-2{\hat{q}}}{n-2}. \end{aligned}$$

From (24) we obtain

25 $$\begin{aligned} {\hat{p}} = \frac{\kappa _- {\hat{q}}}{\kappa _2} {\bar{\alpha }} = \frac{\kappa _- {\hat{q}}(n-2{\hat{q}})}{\kappa _2 (n-2)}, \end{aligned}$$

which is substituted into the sum of (22) and (23):

$$\begin{aligned} (n - 2{\hat{q}})\left( \kappa _1 - \frac{\kappa _1\kappa _-}{\kappa _2(n-2)}{\hat{q}} - \frac{\kappa _{-}^2 (n-1)}{\kappa _2 (n-2)^2}{\hat{q}}^2 - \frac{\kappa _{-1} \kappa _-}{\kappa _2(n-2)}{\hat{q}}\right) = 0. \end{aligned}$$

The option $n=2{\hat{q}}$ leads to ${\bar{\alpha }}=0$, implying ${\hat{p}} = 0$ and, thus, ${\bar{p}}=0$, which contradicts (23). Therefore the second paranthesis has to vanish, leading to a quadratic equation for ${\hat{q}}$ with the only positive solution

$$\begin{aligned} {\hat{q}} = \frac{(n-2)\left( -\kappa _{-1} - \kappa _1 + \sqrt{(\kappa _1 + \kappa _{-1})^2 + 4 \kappa _1 \kappa _2 (n-1)}\right) }{2 \kappa _{-} (n-1)} . \end{aligned}$$

Now (24) implies the formula for ${\bar{\alpha }}$ stated in the theorem and we note that $0<{\bar{\alpha }} <1$ implies $1<{\hat{q}}<n/2$. We compute ${\hat{p}}$ from ${\hat{q}}$ by (25) and note that ${\hat{p}}>0$ since ${\bar{\alpha }}>0$. We then compute ${\hat{s}} = {\bar{s}}/{\bar{r}}=n-{\hat{p}}-2{\hat{q}}$ from the sum of (22) and (23):

$$\begin{aligned} {\hat{s}} = {\hat{p}} \frac{\kappa _{-1}(n-2) +\kappa _- {\hat{q}} (n-1)}{(n-2)\kappa _1} = \frac{\kappa _- {\hat{q}} \left( \kappa _{-1}(n-2) +\kappa _- {\hat{q}} (n-1)\right) }{(n-2)\kappa _1 \kappa _2} {\bar{\alpha }}, \end{aligned}$$

which proves ${\hat{s}}>0$. Finally we obtain the formula for ${\bar{p}}$ from (23) as well as ${\bar{r}}={\bar{p}}/{\hat{p}}$ and ${\bar{q}}={\bar{r}} {\hat{q}}$. $\square$

For convenience below, the conditions in the theorem are made more explicit in terms of the parameters by

26 $$\begin{aligned} {\bar{\alpha }} <1 \;&\Leftrightarrow \; {\hat{q}}>1 \; \Leftrightarrow \; \kappa _1\kappa _2 > \frac{\kappa _-}{n-2}\left( \kappa _1 + \frac{n-1}{n-2}\kappa _- +\kappa _{-1}\right) , \end{aligned}$$

27 $$\begin{aligned} {\bar{\alpha }}>0 \;&\Leftrightarrow \; {\hat{q}}<\frac{n}{2} \; \Leftrightarrow \; \kappa _1\kappa _2 < \frac{\kappa _- n}{2(n-2)}\left( \kappa _1 + \frac{n(n-1)}{2(n-2)} \kappa _-+\kappa _{-1}\right) . \end{aligned}$$

The steady state approaches the origin $p=q=r=0$ as ${\bar{\alpha }} \rightarrow 1$, whereas all its components become unbounded as ${\bar{\alpha }}\rightarrow 0$. This motivates the following.

#### Conjecture 1

With the notation of Theorem 2,

1. if $0<{\bar{\alpha }}<1$, then all solutions of (12) converge to $({\bar{p}},{\bar{q}},{\bar{r}})$ as $t\rightarrow \infty$,
2. if ${\bar{\alpha }}\ge 1$, then all solutions of (12) converge to (0, 0, 0) as $t\rightarrow \infty$,
3. if ${\bar{\alpha }}\le 0$, then for all solutions of (12) we have $p(t),q(t),r(t) \rightarrow \infty$ as $t\rightarrow \infty$.

**Fig. 3:**
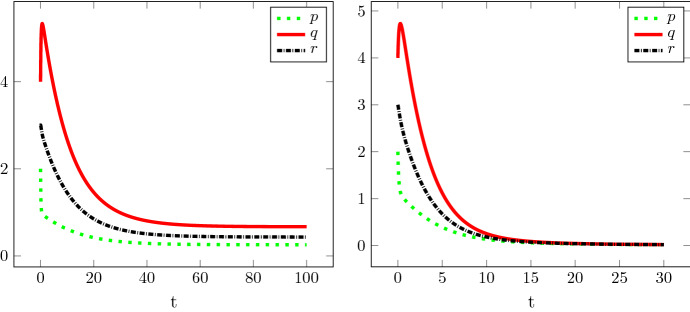
Left: Convergence to the non-trivial steady state of Theorem 2. Simulation of an aggregate (*p*, *q*, *r*) of initial size (2, 4, 3) with parameters $\kappa _1= \kappa _2=\kappa _3=\kappa _{-1}=1$ and $\kappa _{-}=0.6$, implying $0<{\bar{\alpha }}<1$. Right: Instability of the aggregate. Simulation of an aggregate (*p*, *q*, *r*) of initial size (2, 4, 3) with parameters $\kappa _1= \kappa _2=\kappa _3=\kappa _{-1} = 1$ and $\kappa _{-}=0.93$, implying ${\bar{\alpha }}>1$

**Fig. 4:**
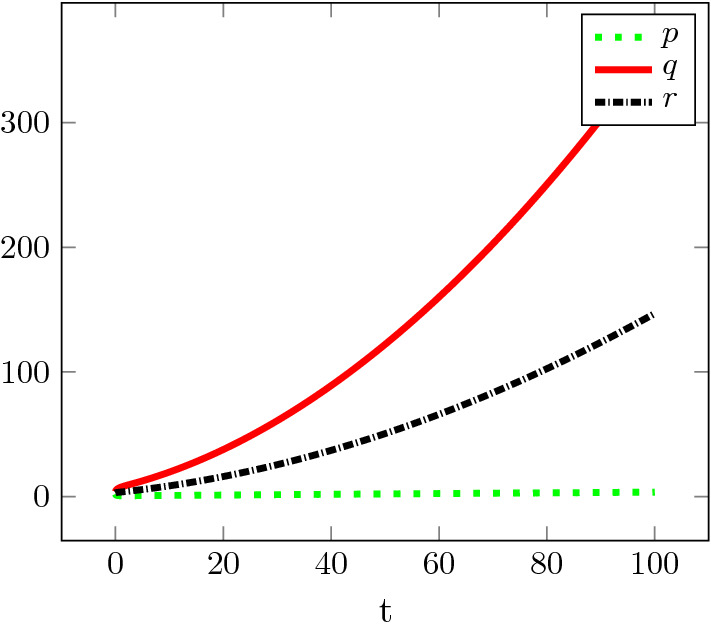
Growth of the aggregate. Simulation of an aggregate (*p*, *q*, *r*) of initial size (2, 4, 3) with parameters $\kappa _1= \kappa _2=\kappa _3=\kappa _{-1} = 1$ and $\kappa _{-}=0.2$, implying ${\bar{\alpha }}<0$

The conjecture has been supported by numerical simulations. Figures 3 and 4 show typical simulation results corresponding to the three cases. Partial rigorous results on the conjecture have been proven in the parallel work (Delacour et al. 2020). These include local stability of the zero steady state for ${\bar{\alpha }} > 1$. Since the right hand side of (12) lacks sufficient smoothness, this result cannot be proven by the standard linearization approach.

Plotting $\log p(t){/}\log t$, $\log q(t){/}\log t$, and $\log r(t){/}\log t$ (not shown) for the solutions depicted in Fig. 4 suggests $\log p(t){/}\log t \rightarrow 1$, $\log q(t){/}\log t, \log r(t){/}\log t \rightarrow 2$ as $t\rightarrow \infty$. This is in agreement with the following formal result.

#### Proposition 1

With the notation of Theorem 2, if ${\bar{\alpha }} < 0$, then there exists a formal approximation of a solution of (12) of the form

28 $$\begin{aligned} p(t)\!=\!p_1 t\!+\! \textit{o}(t),\quad q(t)\!=\!q_2 t^{2}\!+\! \textit{o}(t^{2}),\quad r(t) \!=\! r_2 t^{2}\!+\! \textit{o}(t^{2}),\quad \text{ as } t\rightarrow {\infty },\qquad \qquad \end{aligned}$$

with

29 $$\begin{aligned} \begin{array}{l} p_1 = \frac{\kappa _- n}{\kappa _3 (2n\kappa _2 + \kappa _- n + 4\kappa _{-1})} \left( \kappa _1 \kappa _2 - \frac{\kappa _- n}{2(n-2)} \left( \kappa _1 + \kappa _{-1} + \frac{\kappa _- n(n-1)}{2(n-2)}\right) \right) >0, \\ q_2 = \frac{n}{2}r_2 = \frac{\kappa _3(n-2)(2n\kappa _2 + \kappa _- n + 4\kappa _{-1})}{\kappa _-(4\kappa _1 (n-2) + \kappa _- n^2)} p_1^2. \end{array}\end{aligned}$$

The approximation is (from a formal point of view) unique, including the choice of the exponents of *t*, among solutions with polynomially or exponentially growing aggregate size *r*.

#### Remark

- The precise meaning of the uniqueness statement is best understood from the proof below. The attempt to find an exponentially growing approximation for a solution by formal asymptotic expansions fails. A polynomial ansatz, on the other hand, leads to the result of the theorem, and the exponents and coefficients are determined uniquely by the asymptotics.
- Another result of the parallel work (Delacour et al. 2020) is a rigorous version of Proposition 1. It confirms the existence of solutions with the asymptotic behavior stated in the theorem, and it shows that they are locally attracting. The proof involves advanced techniques from dynamical systems theory such as Poincaré compactification and geometric singular perturbation theory.

#### Proof

In the formal arguments below we shall assume that the solution components behave asymptotically as $t\rightarrow \infty$ either like $e^{\lambda t}$ with $\lambda >0$ or like $t^\gamma$ with $\gamma >0$. This includes the assumption that solution derivatives also behave like the derivatives of these functions.

Since $2r\le 2q \le nr$ holds for admissible states, when *r*(*t*) tends to infinity, then also *q*(*t*) tends to infinity at the same rate, which we write with the *sharp order symbol* $O_s$ as

30 $$\begin{aligned} q(t) = O_s(r(t)) \quad \text{ as } t\rightarrow {\infty }. \end{aligned}$$

With $\alpha = \frac{s+p}{(n-2)r}$, we write the equations for *r* and for $p+q$ as

31 $$\begin{aligned} \dot{r} = \kappa _2 p - (s+p) \frac{\kappa _- q}{(n-2)r},\quad \dot{p} + \dot{q} = \kappa _1 s - p \left( \frac{\kappa _-(n-1)q}{(n-2)r} + \kappa _{-1}\right) . \end{aligned}$$

Since the right hand sides have to be asymptotically nonnegative by the growth of *q* and *r*, taking (30) into account, the first equation implies $s(t) = O(p(t))$, and the second implies $p(t)=O(s(t))$, i.e.

32 $$\begin{aligned} s(t) = O_s(p(t)) \quad \text{ as } t\rightarrow {\infty }. \end{aligned}$$

If the growth were exponential, i.e. $r(t),q(t) \approx c e^{\lambda t}$, $\lambda >0$, then the exponential growth on the left hand sides of (31) would have to be balanced by terms of the same order of magnitude on the right hand sides, i.e. $p(t), s(t) = O_s(e^{\lambda t})$. Then the quadratic negative term $-\kappa _3 p(t)s(t) = O_s(e^{2\lambda t})$ on the right hand side of the first equation in (12) could not be balanced by any of the linear positive terms, and would eventually drive *p* to negative values. This contradiction excludes exponential growth.

For polynomial growth, i.e. $r(t),q(t) \approx c t^\gamma$, the term $\dot{r}(t) \approx c\gamma t^{\gamma -1}$ in (31) needs to be balanced, implying $p(t),s(t) = O_s(t^{\gamma -1})$. In the equation for *q* in (12), $\dot{q}(t), p(t) = O_s(t^{\gamma -1})$ are small compared to $q(t) = O_s(t^\gamma )$. Therefore it is necessary that $s(t)p(t) = O_s(q(t))$, implying $2(\gamma -1)=\gamma$ and, thus, $\gamma =2$. This justifies the ansatz (28) with the addition $s(t) = s_1 t + o(t)$. Substitution into the differential equations and comparison of the leading-order terms gives equations for the coefficients:

$$\begin{aligned} \text{2nd } \text{ equ. } \text{ in } (12): \quad&0 = \kappa _3 p_1 s_1 - \kappa _- q_2,\\ (17): \quad&0 = \kappa _3 p_1 s_1 - \kappa _- q_2 \left( 1 - \alpha (q_2,r_2)\right) ,\\ \text{1st } \text{ equ. } \text{ in } (31): \quad&2r_2 = \kappa _2 p_1 - (s_1+p_1) \frac{\kappa _- q_2}{(n-2)r_2}, \\ \text{2nd } \text{ equ. } \text{ in } (31): \quad&2q_2 = \kappa _1 s_1 - p_1 \left( \frac{\kappa _-(n-1)q_2}{(n-2)r_2} + \kappa _{-1}\right) , \end{aligned}$$

This system can be solved explicitly by first noting that the first two equations imply $\alpha (q_2,r_2)=0$ and, thus, $2q_2=nr_2$. Using this in the third and fourth equation gives a linear relation between $p_1$ and $s_1$. This again can be used in the fourth equation to write $q_2$ as a linear function of $s_1$. The division of the first equation by $s_1$ then gives the formula for $p_1$ in (29). The positivity of $p_1$ is a consequence of (27). $\square$

For all the results so far the positivity of the rate constant $\kappa _-$ for breaking cross-links has been essential. Therefore it seems interesting to consider the special case $\kappa _- = 0$ separately. It turns out that the dynamics is much simpler. The aggregate size always grows linearly with time.

#### Theorem 3

Let $3\le n\in {\mathbb {N}}$, $\kappa _1,\kappa _2,\kappa _3,\kappa _{-1}>0$, and $\kappa _{-} = 0$. Let $(p_0,q_0,r_0) \in (0,\infty )^3$ satisfy (14). Then the solution of (12) satisfies

$$\begin{aligned}&\lim _{t\rightarrow \infty }p(t) = p_{\infty }:= \frac{(n-2) \kappa _1 \kappa _2}{\kappa _3 ( \kappa _2 (n-2) + \kappa _{-1})} ,\qquad \lim _{t\rightarrow \infty }s(t) = s_{\infty }:= \frac{(n-2) \kappa _2}{2 \kappa _3} ,\\&q(t) = p_\infty (\kappa _2 + \kappa _3 s_\infty )t + o(t) ,\qquad r(t) = \kappa _2 p_\infty t + o(t) , \qquad \text{ as } t\rightarrow \infty . \end{aligned}$$

#### Proof

For $\kappa _{-} = 0$ the right hand sides in (12) depend only on *p* and $s=nr-2q-p$, meaning that these two variables solve a closed system:

$$\begin{aligned} \dot{p}&= \kappa _1 s - (\kappa _2 + \kappa _{-1} + \kappa _3 s)p ,\\ \dot{s}&= ((n-1)\kappa _2 + \kappa _{-1})p - (\kappa _1 + \kappa _3 p)s . \end{aligned}$$

The unique nontrivial steady state $(p_{\infty },s_{\infty })$ can be computed explicitly. We prove that it is globally attracting by constructing a Lyapunov functional. Let $a\ge 1$ and

$$\begin{aligned} R_a := \left[ \frac{p_\infty }{a}, ap_\infty \right] \times \left[ \frac{s_\infty }{a}, as_\infty \right] . \end{aligned}$$

For each point $(p,s)\in (0,\infty )^2$ there is a unique value of $a\ge 1$ such that $(p,s)\in \partial R_a$. Therefore the Lyapunov function

$$\begin{aligned} L(p,s) := a-1 \qquad \text{ for } (p,s) \in \partial R_a , \end{aligned}$$

is well defined and definite in the sense $L(p,s)\ge 0$ with equality only for $(p,s)=(p_\infty ,s_\infty )$. It remains to prove that the flow on $\partial R_a$ is strictly inwards. For example, for the left boundary part,

$$\begin{aligned} \dot{p}\bigm |_{p=p_\infty /a}= & {} (\kappa _2 + \kappa _{-1} + \kappa _3 s)\left( \frac{\kappa _1 s}{\kappa _2 + \kappa _{-1} + \kappa _3 s} - \frac{p_\infty }{a}\right) \\\ge & {} \frac{1}{a}(\kappa _2 + \kappa _{-1} + \kappa _3 s)\left( \frac{\kappa _1 s_\infty }{\kappa _2 + \kappa _{-1} + \kappa _3 s_\infty /a} - p_\infty \right) >0 , \end{aligned}$$

where the first inequality follows from $s\ge s_\infty /a$, and the second inequality from $a>1$ and from the fact that the last parenthesis vanishes for $a=1$, since $(p_\infty ,s_\infty )$ is an equilibrium. Analogously, for the right boundary part,

$$\begin{aligned} \dot{p}\bigm |_{p=a p_\infty }= & {} (\kappa _2 + \kappa _{-1} + \kappa _3 s)\left( \frac{\kappa _1 s}{\kappa _2 + \kappa _{-1} + \kappa _3 s} - a p_\infty \right) \\\le & {} a(\kappa _2 + \kappa _{-1} + \kappa _3 s) \left( \frac{\kappa _1 s_\infty }{\kappa _2 + \kappa _{-1} + \kappa _3 a s_\infty } - p_\infty \right) < 0 , \end{aligned}$$

for the top boundary part,

$$\begin{aligned} \dot{s}\bigm |_{s=a s_\infty }= & {} (\kappa _1 + \kappa _3 p)\left( \frac{((n-1)\kappa _2 + \kappa _{-1})p}{\kappa _1 + \kappa _3 p} - a s_\infty \right) \\\le & {} a(\kappa _1 + \kappa _3 p)\left( \frac{((n-1)\kappa _2 + \kappa _{-1})p_\infty }{\kappa _1 + \kappa _3 a p_\infty } - s_\infty \right) <0 , \end{aligned}$$

and for the bottom boundary part,

$$\begin{aligned} \dot{s}\bigm |_{s=s_\infty /a}= & {} (\kappa _1 + \kappa _3 p)\left( \frac{((n-1)\kappa _2 + \kappa _{-1})p}{\kappa _1 + \kappa _3 p} - \frac{s_\infty }{a}\right) \\\ge & {} \frac{1}{a}(\kappa _1 + \kappa _3 p)\left( \frac{((n-1)\kappa _2 + \kappa _{-1})p_\infty }{\kappa _1 + \kappa _3 p_\infty /a} - s_\infty \right) >0 . \end{aligned}$$

This shows that the Lyapunov functional is strictly decreasing along solutions, completing the proof of global asymptotic stability of the equilibrium.

The linear growth of *q* and *r* follows from

$$\begin{aligned} \lim _{t\rightarrow \infty } \dot{q}(t) = \kappa _2 p_\infty + \kappa _3 p_\infty s_\infty ,\qquad \lim _{t\rightarrow \infty }\dot{r}(t) = \kappa _2 p_\infty . \end{aligned}$$

$$\square $$

This result shows that the breakage of cross-links has somewhat contradictory effects, depending on the parameter regime: It can speed-up the aggregation dynamics, producing a quadratic rather than linear growth of the aggregate size (Case 3 of Conjecture 1 and Proposition 1). This is linked to the fact that it allows the aggregates to rearrange in a more compact way. On the other hand, it may slow down the dynamics, such that the aggregate only reaches a finite size (Case 1) or even disintegrates completely (Case 2), again compared to the linear growth without cross-link breaking (Theorem 3).

## Comparison with experimental data: discussion

### Comparison with experimental data

**Fig. 5:**
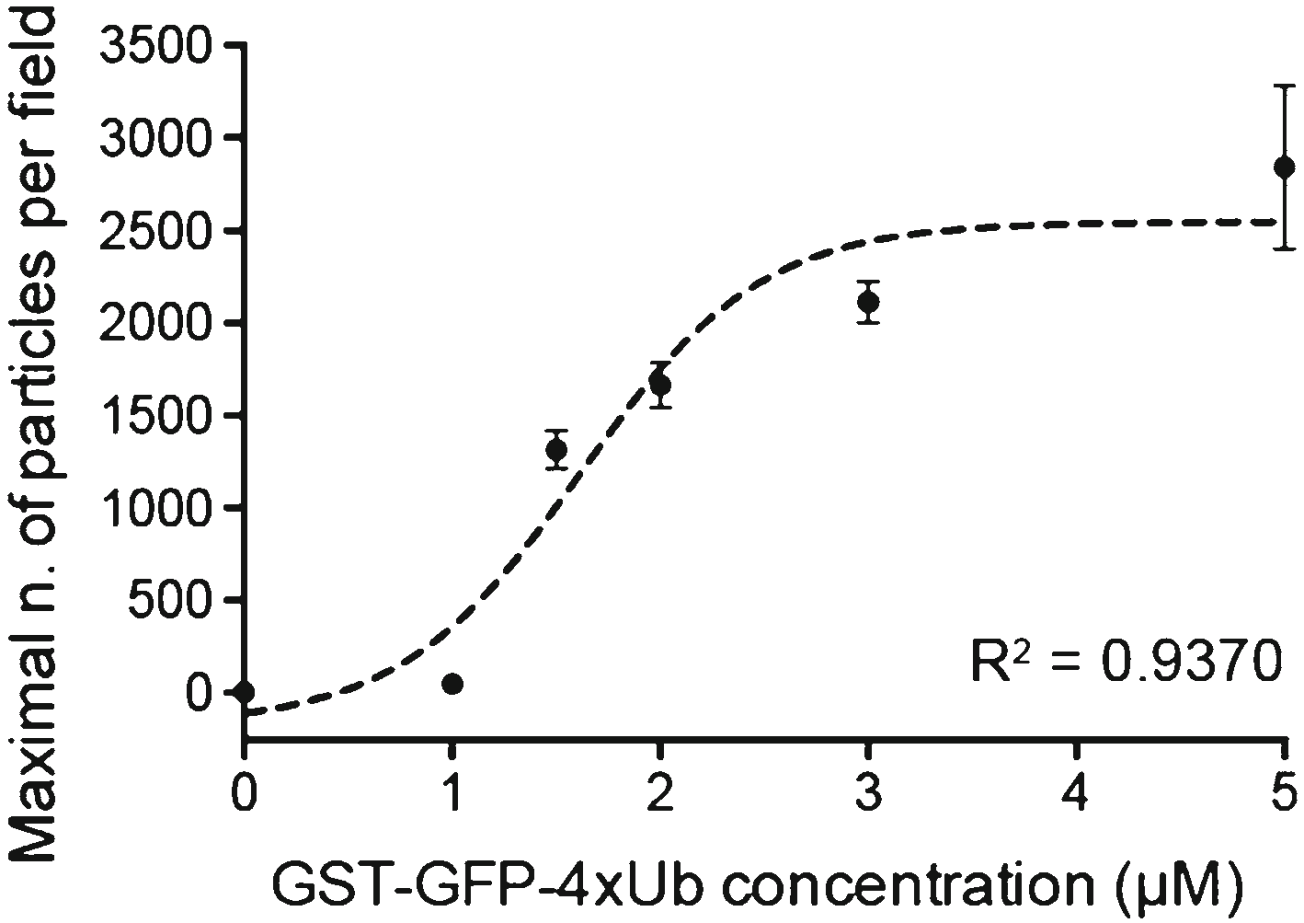
Number of aggregates in terms of [*Ubi*] (or more precisely $(4\times Ubi-GST-GFP)_2$) at fixed $[{\mathrm{p}}62]=2\,\upmu$M (Zaffagnini et al. 2018). Average and SD among three independent replicates are shown. The dashed line represents a fitted sigmoidal (more precisely, logistic) function, centered around $[Ubi] = 1.6\,\upmu$M. Note that here p62 monomers are counted. Under the assumption that p62 only occurs in oligomers of size *n* we have [p62] = *n*[p62$_n$]. The regression coefficient R$^2$ measures the quality of the fit

There are only limited options for a serious comparison of the theoretical results with experimental data. We shall use the data shown in Fig. 5, which have been published in Zaffagnini et al. (2018). It provides observed numbers of aggregates in dependence of ubiquitin for a fixed concentration of p62. Our results do not permit a direct comparison with this curve, which would require modelling of the process of nucleation of aggregates. However, the data provide at least some information about concentration levels of ubiquitin and p62, such that stable aggregates exist.

For meaningful quantitative comparisons with these scarce data we need to reduce the number of parameters in our model. As a first step, we fix the value $n=5$ of the size of p62 oligomers, following Zaffagnini et al. (2018) where values between 5 and 6 for GFP-p62 have been found (although we note that in Wurzer et al. (2015) an average of about $n=24$ has been reported for mCherry-p62 in vitro). Since the experiment in Zaffagnini et al. (2018) has been carried out with a fixed p62 concentration $[\mathrm{p62}] = 2\,\upmu$M, this corresponds to an oligomer concentration of $[\mathrm{p62}_5] = [\mathrm{p62}]/5 = 0.4\,\upmu$M.

Concerning the rate constants, we make the assumption that the binding and, respectively, the unbinding rate constants are equal, i.e. $\kappa _1' = \kappa _2'$ and $\kappa _{-1}=\kappa _-$. This will allow to express all our results in terms of one *dissociation constant* $K_d := \kappa _{-1}/\kappa _1'$.

From Fig. 5 we conclude that for an oligomer concentration of $[\mathrm{p62}_5] = 0.4\,\upmu$M the growth of stable aggregates requires a minimal cross-linker concentration [*Ubi*] roughly between 0.6 and $2.6\,\upmu$M ($(1.6\pm 1)\,\upmu$M, where $1.6\,\upmu$M and $1\,\upmu$M are rough estimates of the inflection point and, respectively, the spread of the curve). According to the results of the preceding section, these values should correspond to situations with either ${\bar{\alpha }}=0$ or ${\bar{\alpha }}=1$, depending on the question, if the equilibrium aggregate sizes of Case 1 in Conjecture 1 are large enough to be detected in the experiment, or if we need to be in Case 3 of growing aggregates. Therefore, with the above assumptions, with $\kappa _1 = \kappa _1' [Ubi]$, $\kappa _2 = \kappa _2' [\mathrm{p62}_5]$, and with (26), (27), we obtain for ${\bar{\alpha }}=1$:

33 $$\begin{aligned}{}[\mathrm{p62}_n]\,[Ubi] = \frac{K_d}{n-2} \left( [Ubi] + \frac{(2n-3)K_d}{n-2}\right) , \end{aligned}$$

and for $${\bar{\alpha }} = 0$$:

34 $$\begin{aligned}{}[\mathrm{p62}_n]\,[Ubi] = \frac{n K_d}{2(n-2)} \left( [Ubi] + \frac{(n^2 + n - 4)K_d}{2(n-2)}\right) . \end{aligned}$$

Solving these equations for $K_d$ with $n=5$, $[\mathrm{p62}_n] = 0.4\,\upmu$M, and with [*Ubi*] between $0.6\,\upmu$M and $2.6\,\upmu$M, gives estimates for $K_d$ between $0.44\,\upmu$M and $0.73\,\upmu$M for ${\bar{\alpha }}=1$, and between $0.20\,\upmu$M and $0.31\,\upmu$M for ${\bar{\alpha }}=0$. So we claim that at least the order of magnitude is significant. It differs by three orders of magnitude from published data on the reaction between ubiquitin and the UBA domain of p62 [$K_d \approx 540\,\upmu$M (Long et al. 2010)]. This should not be so surprising, since in the context of growing aggregates the reactions can be strongly influenced by avidity effects.

### Discussion

**Fig. 6:**
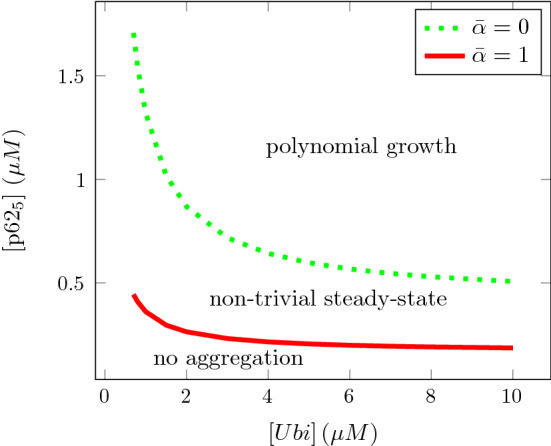
Bifurcation diagram corresponding to Conjecture 1 for $n=5$, $K_d = 0.5\,\upmu$M with the bifurcation curves given by (33) and (34)

We return to Conjecture 1, where the long-time behaviour is described in terms of the value of the parameter ${\bar{\alpha }}$ defined in (20). With the simplifying assumptions on the reaction rate constants from above, the statements of the conjecture are depicted in Fig. 6 for the fixed values $n=5$ and $K_d = 0.5\,\upmu$M (motivated by the estimates above) in a bifurcation diagram in terms of the concentrations [*Ubi*] and $[\mathrm{p62}_n]$. In the derivation of the model we have stated the assumption that [*Ubi*] and $[\mathrm{p62}_n]$ are of the same order of magnitude, meaning that their ratio has been kept fixed when taking asymptotic limits ($k_0, t\rightarrow \infty$). The bifurcation diagram gives some indication of what happens when they take extreme values. The right hand sides of (33) and (34) indicate that smallness of [*Ubi*] or $[\mathrm{p62}_n]$ can be compensated by largeness of the other, to obtain stable or growing aggregates. However, the occurrence of [*Ubi*] on the right hand sides produces an unsymmetry. If $[\mathrm{p62}_n] < \frac{K_d}{n-2}$ aggregates are unstable, no matter how large [*Ubi*] is. Similarly, polynomial growth never happens for $[\mathrm{p62}_n] < \frac{n K_d}{2(n-2)}$.

**Fig. 7:**
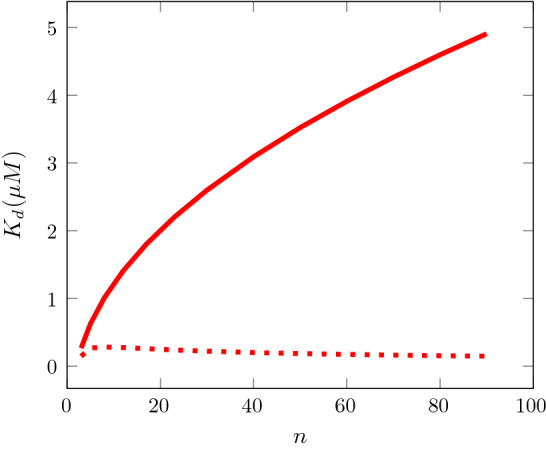
The dissociation constant $K_d$ determined from (33) (solid line) and (34) (dashed line), depending on the p62 oligomer size *n*. Ubiquitin and p62 oligomer concentrations from Fig. 5 at the onset of aggregation: $[Ubi]=1.6\,\upmu$M, $[\mathrm{p62}_n]=0.4\,\upmu$M

There is a significant uncertainty concerning the oligomer size *n*, which has so far been assumed to be 5, according to observations in Zaffagnini et al. (2018). Actually, a distribution of oligomer sizes should be expected in the experiments of Fig. 5 with the occurrence of much larger oligomers. An extension of our model to a situation with a given size distribution of free oligomers would be feasible. Because of the fact that the rate of adding oligomers to the aggregate is independent of the oligomer size [see (3)] it would then be reasonable to assume that also within the aggregate the relative oligomer size distribution is the same as for free oligomers. Without having carried out the computations in detail, we expect a model of the same form as ours, where quantities depending on the oligomer size have to be replaced by suitably computed mean values. There does not seem to be any reason for a different qualitative behavior of the modified model. As an indication of the effects of varying the oligomer size the computation of $K_d$ from (33) has been repeated for a range of values of *n* between $n=3$ and $n=100$. The results are depicted in Fig. 7, which shows that the predicted values of $K_d$ might be larger by up to an order of magnitude compared to the case $n=5$, but still small compared to Long et al. (2010), if larger oligomer sizes are considered and ${\bar{\alpha }}=1$ is relevant. The asymptotic behaviour for large oligomer sizes is easily seen to be $K_d = O(n^{1/2})$. On the other hand, if ${\bar{\alpha }}=0$ is relevant, the value of $K_d$ becomes smaller by up to an order of magnitude for large oligomers with the asymptotic behaviour $K_d= O(n^{-1/2})$. These asymptotic behaviors are easily seen from the explicit solutions of the quadratic equations (33) and (34).

## Conclusion

In this article, we have proposed an ODE model for the growth and decay of aggregates of p62 oligomers cross-linked by ubiquitin chains. Under the assumption of unlimited supply of free oligomers and cross-linkers we found three possible asymptotic regimes: complete degradation of aggregates, convergence towards a finite aggregate size, and unlimited growth (quadratic in time) of the aggregate size. In the latter case, growing aggregates are asymptotically tightly packed with the maximum number of cross-links. These statements are supported by a mixture of explicit steady state computations, formal asymptotic analysis, and numerical simulations. The three regimes, which can be separated explicitly in terms of the reaction constants, have been illustrated by the simulation results. Rigorous proofs of some results on the long-time behaviour in the three regimes are the subject of the parallel work (Delacour et al. 2020).

A comparison of the theoretical results with data from Zaffagnini et al. (2018) has provided an estimate for the dissociation constant of the elementary reaction between ubiquitin and the UBA domain of p62 in the context of growing aggregates.

There are several possible extensions of this work. A limitation of the original discrete model is that the description of aggregates by triplets (*i*, *j*, *k*) is very incomplete. Typically, very different configurations are described by the same triplet. For example, we could imagine very homogeneous or very heterogeneous aggregates, i.e. fully packed in certain regions and very loose in others. Reaction rates will strongly depend on the configuration, including information about the geometry of the aggregate. In principle one can imagine an attempt to overcome these difficulties based on a random graph model (Frieze and Karonski 2015), but the resulting model describing probability distributions on the sets of all possible aggregate shapes would be prohibitively complex. An intermediate solution would be a more serious approach to finding formulas for quantities like the probability $\alpha$ of losing an oligomer, when a cross-link breaks, based on typical probability distributions.

The model (12) describes an intermediate stage of the aggregation process. On the one hand, the large aggregate assumption means that we are dealing with the growth of already developed aggregates, neglecting the nucleation process, which is important for the number of established aggregates. A model of the nucleation process would be based on the discrete representation and it would have to be stochastic. On the other hand, we neglect two effects important for a later stage of the process. The first and obvious one is the limited availability of free p62 oligomers and ubiquitin cross-linkers. It would be rather straightforward to incorporate this into the model, however at the expense of increased complexity. It would also eliminate the dichotomy between the Cases 1 and 3 of Conjecture 1 since unbounded growth would be impossible. For relatively large initial concentrations of free particles, one could imagine a two-time-scale behaviour with an initial quadratic growth and saturation on a longer time scale. The other effect, which is neglected here but definitely present in experiments, is coagulation of aggregates. This is the subject of ongoing work, based on the PDE model (38) derived in the Appendix and enriched by an account of the coagulation process. A more complete model for a distribution of aggregates, including nucleation and coagulation, would permit a more substantial comparison with experiments as in Zaffagnini et al. (2018), whose output should be extended to provide information about aggregate size distributions. A new experimental challenge suggested by our model is to detect the oligomer-to-crosslinker ratio in aggregates, which could be compared to our theoretical predictions.

More generally, it is very common that protein aggregates contain different types of molecules. In particular, the occurrence of ubiquitinated aggregates has been observed in *pathological conditions* [e.g. in neurodegenerative diseases (Donaldson et al. 2003) or in diabetes (Kaniuk et al. 2007)]. Existing mathematical models of these aggregation processes (see, e.g. Prigent et al. 2014) could be extended by the approach of this work. Finally, various cytoskeletal structures can be seen as heterogeneous protein aggregates, for example *actin filament networks* with filamin, fascin, or Arp2/3 complex as cross-linkers (Vinzenz et al. 2012), or *focal adhesions,* containing integrins, talin, vinculin, and several other protein species (Zamir and Geiger 2001). The latter seems particularly interesting because of the dependence of the growth behaviour on mechanical stimuli (Riveline et al. 2001). Again the approach of this work might provide an alternative basis for mathematical modeling.

## Large aggregate limit

We denote by $c_{i,j,k}(t)$ the probability of the aggregate to be in the state (*i*, *j*, *k*) at time *t*. Its evolution will be determined by a jump process model of the reactions with the rates given in (2), (3), (4), (5), (6), (7), (9), and (10).

For this purpose the relation between pre-reaction state $(i',j',k')$ and post-reaction state (*i*, *j*, *k*) needs to be inverted. A simple example is Reaction 1, i.e. $(i,j,k)=(i'+1,j',k')$, with the inversion $(i',j',k')=(i-1,j,k)$. Using (2), the equation for $c_{i,j,k}$ will therefore contain the source term $(r_1 c)_{i-1,j,k} = \kappa _1(nk-i+1-2j)c_{i-1,j,k}$.

If the unique inversion fails, the source term is a sum over all pre-reaction states producing the post-reaction state (*i*, *j*, *k*). This has to be considered for Reaction 5, where we have $j=j'-1$, $k=k'-1$, and, with (9),

35 $$\begin{aligned} i = i' + 1 - \ell _{i',j',k'} = i' + 1 - \left\lfloor \frac{(n-1)i'}{nk'-2j'} \right\rceil . \end{aligned}$$

The inversion is not possible in general. Occasionally, $\ell _{i',j',k'}$ will increase by one, when $i'$ is increased by one, implying that *i* might take the same value for two consecutive values of $i'$. Even worse: For the extreme case $nk'-2j'=n-1$, where after the loss of a p62 oligomer all binding sites are busy with two-hand bound *Ubi* except the one remaining after breaking the connection, i.e. $nk-2j=1=i$. This state is independent from the number $i'\in \{0,\ldots ,n-1\}$ of one-hand bound *Ubi* getting lost with the oligomer. Therefore we introduce the set of pre-collisional values

$$\begin{aligned} I_{i,j,k} = \left\{ i':\, i = i' + 1 - \ell _{i',j+1,k+1}\right\} \end{aligned}$$

The equation for the probability distribution reads

36 $$\begin{aligned} \frac{dc_{i,j,k}}{dt}= & {} (r_1c)_{i-1,j,k} - (r_1c)_{i,j,k} + (r_2c)_{i+1,j-1,k-1} - (r_2c)_{i,j,k}\nonumber \\&+ (r_3c)_{i+1,j-1,k} - (r_3c)_{i,j,k} \nonumber \\&+\, (r_{-1}c)_{i+1,j,k} - (r_{-1}c)_{i,j,k} + \sum _{i'\in I_{i,j,k}} (r_{-2}c)_{i',j+1,k+1} - (r_{-2}c)_{i,j,k} \nonumber \\&+\, (r_{-3}c)_{i-1,j+1,k} - (r_{-3}c)_{i,j,k}. \end{aligned}$$

We introduce a typical value $k_0$ for the number *k* of oligomers in the aggregate and use it also as a reference value for *i* and *j*, leading by the definition (11) to the scaled triplet (*p*, *q*, *r*). The latter lives on a grid with spacing $\Delta p = \Delta q = \Delta r := 1/k_0$ and, thus, becomes a continuous variable in the large aggregate limit $k_0\rightarrow \infty$. Therefore we postulate the existence of a probability density *P*(*p*, *q*, *r*, *t*) such that

$$\begin{aligned} c_{i,j,k}(t) \approx k_0^{-3}\, P\left( \frac{i}{k_0}, \frac{j}{k_0}, \frac{k}{k_0}, t\right) . \end{aligned}$$

The ansatz above is motivated by

37 $$\begin{aligned} 1= & {} \sum _{(i,j,k)\in {\mathcal {A}}_{d}} c_{i,j,k} = \sum _{(i,j,k)\in {\mathcal {A}}_d} P(i\Delta p,j\Delta q,k\Delta r,t)\Delta p\,\Delta q\,\Delta r\nonumber \\&{\mathop {\longrightarrow }\limits ^{k_0\rightarrow \infty }} \int _{{\mathcal {A}}} P(p,q,r,t) d(p,q,r) , \end{aligned}$$

showing that *P* is a probability density on the continuous set of admissible states ${\mathcal {A}}$, defined by (14). For the set of discrete admissible states defined by (1) we have used the notation ${\mathcal {A}}_d$. Multiplication of (36) by $k_0^3$ and the limit $k_0\rightarrow \infty$ ($\Delta p = \Delta q = \Delta r \rightarrow 0$) will lead to an equation for *P*. This formal procedure would be hard to justify rigorously. An alternative is to write a ’weak’ formulation of (36) by multiplication with a smooth test function $\phi _{i,j,k} = \phi \left( \frac{i}{k_0}, \frac{j}{k_0}, \frac{k}{k_0}\right)$ and summation over $(i,j,k)\in {\mathcal {A}}_d$. The trick is to avoid the appearance of derivatives of *P* in the limit by first shifting the differences in (36) to the test function by ’summation by parts’. In the limit the sums become integrals [as in (37)] and the limiting equation is obtained by an integration by parts. We are confident that this latter procedure could be rigorously justified. However, the necessary estimates and functional analytic framework would go beyond the scope of this article.

We deal with the six differences on the right hand side of (36), corresponding to the six reactions, separately.

### Reaction 1

As an illustration we outline both procedures described above for this simple case, and start with the direct limit:

$$\begin{aligned}&k_0^3 \left[ (r_1c)_{i-1,j,k} - (r_1c)_{i,j,k}\right] \\&\quad \approx \frac{1}{\Delta p} \left[ \kappa _1(nr-p+\Delta p - 2q)P(p-\Delta p,q,r,t) - \kappa _1 (nr-p-2q)P(p,q,r,t)\right] \\&\qquad \rightarrow -\partial _p (\kappa _1(nr-p-2q)P) . \end{aligned}$$

On the other hand,

$$\begin{aligned}&\sum _{(i,j,k)\in {\mathcal {A}}_d} \left[ (r_1c)_{i-1,j,k} - (r_1c)_{i,j,k}\right] \phi _{i,j,k} = \sum _{(i,j,k)\in {\mathcal {A}}_d} (r_1c)_{i,j,k} \left( \phi _{i+1,j,k} - \phi _{i,j,k}\right) \\&\sum _{(i,j,k)\in {\mathcal {A}}_d} \kappa _1 (nr-p-2q)P(p,q,r,t) \frac{\phi (p+\Delta p,q,r) - \phi (p,q,r)}{\Delta p} \Delta p\,\Delta q\,\Delta r \\&\quad \rightarrow \int _{{\mathcal {A}}} \kappa _1(nr-p-2q)P \partial _p \phi \,d(p,q,r)\\&\quad = -\int _{{\mathcal {A}}} \partial _p(\kappa _1(nr-p-2q)P) \phi \,d(p,q,r), \end{aligned}$$

confirming the result above.

### Reaction 2

$$\begin{aligned}&k_0^3 \left[ (r_2c)_{i+1,j-1,k-1} - (r_2c)_{i,j,k} \right] \\&\quad \approx \frac{1}{\Delta p} \left[ \kappa _2(p+\Delta p)P(p+\Delta p,q-\Delta q,r-\Delta r,t)- \kappa _2 p P(p,q-\Delta q,r-\Delta r,t)\right] \\&\qquad +\, \frac{1}{\Delta q} \left[ \kappa _2p P(p,q-\Delta q,r-\Delta r,t) - \kappa _2 p P(p,q,r-\Delta r,t)\right] \\&\qquad + \frac{1}{\Delta r} \left[ \kappa _2p P(p,q,r-\Delta r,t) - \kappa _2 p P(p,q,r,t)\right] \\&\quad \rightarrow \partial _p (\kappa _2 pP) - \partial _q (\kappa _2 pP) - \partial _r (\kappa _2 pP). \end{aligned}$$

### Reaction 3

As explained in Sect. 2, we set $\kappa _3' = \kappa _3/k_0$ and assume that $\kappa _3$ takes moderate values, i.e. we keep it fixed as $k_0\rightarrow \infty$.

$$\begin{aligned}&k_0^3 \left[ (r_3c)_{i+1,j-1,k} - (r_3c)_{i,j,k} \right] \\&\quad \approx \frac{1}{\Delta p} \bigl [ \kappa _3(p+\Delta p)(nr-p-\Delta p-2q+2\Delta q)P(p+\Delta p,q-\Delta q,r,t) \\&\qquad - \kappa _3 p(nr-p-2q+2\Delta q) P(p,q-\Delta q,r,t)\bigr ] \\&\qquad + \frac{1}{\Delta q} \left[ \kappa _3 p(nr-p-2q+2\Delta q) P(p,q-\Delta q,r,t) - \kappa _3 p(nr-p-2q) P(p,q,r,t)\right] \\&\quad \rightarrow \partial _p (\kappa _3 p(nr-p-2q)P) - \partial _q (\kappa _3 p(nr-p-2q)P) . \end{aligned}$$

### Reaction 4

$$\begin{aligned}&k_0^3 \left[ (r_{-1}c)_{i+1,j,k} - (r_{-1}c)_{i,j,k} \right] \\&\quad \approx \frac{1}{\Delta p} \left[ \kappa _{-1}(p+\Delta p)P(p+\Delta p,q,r,t) - \kappa _{-1} p P(p,q,r,t)\right] \\&\quad \rightarrow \partial _p (\kappa _{-1} pP) . \end{aligned}$$

### Reaction 5

In this case it is actually simpler to proceed via the weak formulation. As a preparatory step, we recall that $i'\in I_{i,j,k}$ iff $i = i' + 1 - \ell _{i',j+1,k+1}$ with

$$\begin{aligned} \ell _{i,j,k} = \left\lfloor \frac{(n-1)i}{nk-2j}\right\rceil = \left\lfloor \frac{(n-1)p}{nr-2q}\right\rceil =: \ell (p,q,r) . \end{aligned}$$

We shall also need

$$\begin{aligned} \alpha _{j,k} \rightarrow \frac{nr-2q}{(n-2)r} =: \alpha (q,r) . \end{aligned}$$

In the following computation the summation of gain terms is renumbered from $(i,j,k)\in {\mathcal {A}}_d$ to $(i',j',k')\in {\mathcal {A}}_d$ with $i'\in I_{i,j,k}$, $j' = j+1$, $k'=k+1$:

$$\begin{aligned}&\sum _{(i,j,k)\in {\mathcal {A}}_d} \left( \sum _{i'\in I_{i,j,k}} (r_{-2}c)_{i',j+1,k+1} - (r_{-2}c)_{i,j,k}\right) \phi _{i,j,k} \\&\quad = \sum _{(i,j,k)\in {\mathcal {A}}_d} (r_{-2}c)_{i,j,k} \left( \phi _{i+1-\ell _{i,j,k},j-1,k-1} - \phi _{i,j,k}\right) \\&\quad = \sum _{(i,j,k)\in {\mathcal {A}}_d} \kappa _- \alpha _{j,k} q (\ell -1)P\\&\qquad \frac{\phi (p-\Delta p(\ell -1),q-\Delta q,r-\Delta r) - \phi (p,q-\Delta q,r-\Delta r)}{\Delta p(\ell -1)} \Delta p\,\Delta q\,\Delta r \\&\qquad + \sum _{(i,j,k)\in {\mathcal {A}}_d} \kappa _- \alpha _{j,k} q P\frac{\phi (p,q-\Delta q,r-\Delta r) - \phi (p,q,r-\Delta r)}{\Delta q} \Delta p\,\Delta q\,\Delta r \\&\qquad + \sum _{(i,j,k)\in {\mathcal {A}}_d} \kappa _- \alpha _{j,k} q P\frac{\phi (p,q,r-\Delta r) - \phi (p,q,r)}{\Delta r} \Delta p\,\Delta q\,\Delta r \\&\quad \rightarrow - \int _{{\mathcal {A}}} \kappa _- \alpha q P \left( (\ell -1)\partial _p \phi + \partial _q \phi + \partial _r \phi \right) d(p,q,r) \\&\quad = \int _{{\mathcal {A}}} \left( \partial _p (\kappa _- \alpha q(\ell -1) P) + \partial _q(\kappa _- \alpha q P) + \partial _r(\kappa _- \alpha q P)\right) \phi \, d(p,q,r). \end{aligned}$$

Finally we introduce a simplification by dropping the rounding operation in $\ell$. This avoids a technical difficulty with a lack of smoothness, but most likely it does not change the qualitative properties of the model.

### Reaction 6

$$\begin{aligned}&k_0^3 \left[ (r_{-3}c)_{i+1,j-1,k} - (r_{-3}c)_{i,j,k} \right] \\&\quad \approx \frac{1}{\Delta p} \bigl [ \kappa _- (1-\alpha (q+\Delta q,r))(q+\Delta q)P(p-\Delta p,q+\Delta q,r,t) \\&\qquad - \kappa _- (1-\alpha (q+\Delta q,r))(q+\Delta q) P(p,q+\Delta q,r,t)\bigr ] \\&\qquad + \frac{1}{\Delta q} \Big [ \kappa _- (1-\alpha (q+\Delta q,r))(q+\Delta q) P(p,q+\Delta q,r,t)\\&\qquad - \kappa _- (1-\alpha (q,r))q P(p,q,r,t)\Big ] \\&\quad \rightarrow -\partial _p (\kappa _- (1-\alpha )qP) + \partial _q (\kappa _- (1-\alpha )qP) . \end{aligned}$$

Collecting our results, the limiting equation for the evolution of *P* reads

38 $$\begin{aligned}&\partial _t P + \partial _p \left( \left( (\kappa _1 - \kappa _3 p)(nr-p-2q) - (\kappa _2 + \kappa _{-1})p + \kappa _- q \left( 1 - \frac{(n-1)p}{(n-2)r}\right) \right) P\right) \nonumber \\&\quad + \partial _q \left( (\kappa _2 p+\kappa _3 p(nr-p-2q) - \kappa _- q)P\right) + \partial _r \left( (\kappa _2 p - \kappa _- \alpha q)P\right) = 0 . \end{aligned}$$

For deterministic initial conditions of the form $P(p,q,r,0) = \delta (p-p_0)\delta (q-q_0)\delta (r-r_0)$ the state remains deterministic: $P(p,q,r,t) = \delta (p-p(t))\delta (q-q(t))\delta (r-r(t))$, where (*p*(*t*), *q*(*t*), *r*(*t*)) solves the initial value problem (12).
